# Turner syndrome and postpubertal Empty sella syndrome: a case report and literature review

**DOI:** 10.3389/fendo.2025.1552724

**Published:** 2025-06-03

**Authors:** Fanyu Lin, Jing Zeng

**Affiliations:** ^1^ Department of General Internal Medicine, West China Second University Hospital, Sichuan University, Chengdu, Sichuan, China; ^2^ Key Laboratory of Birth Defects and Related Diseases of Women and Children (Sichuan University), Ministry of Education, Chengdu, Sichuan, China

**Keywords:** Turner syndrome, Empty sella syndrome, multiple hypopituitarism, central hypothyroidism, hypogonadotropic hypogonadism

## Abstract

**Introduction:**

Turner syndrome is a common sex chromosome disorder characterized by short stature, gonadal dysgenesis, and hypergonadotropic hypogonadism. Empty Sella Syndrome is defined radiologically as the presence of cerebrospinal fluid filling the sella turcica and is associated with hypopituitarism. The association between TS and ESS is rare.

**Methods:**

We present a case of Turner syndrome associated with Empty Sella Syndrome, characterized by complete development of secondary sexual characteristics but irregular menstruation. Magnetic resonance imaging of the pituitary gland revealed partial empty sella with evidence of pituitary compression. Laboratory investigations indicated hypogonadotropic hypogonadism, central hypothyroidism, and an insulin-induced hypoglycemia test suggested insufficient compensatory growth hormone secretion, while cortisol compensation was normal. To ascertain the correlation between Turner syndrome and Empty Sella Syndrome, we reviewed the literature and tried to explore the potential pathophysiological mechanisms underlying their co-occurrence, thereby providing evidence and reference value for clinical diagnosis.

**Discussion:**

The etiology of post-pubertal pituitary dysfunction in patients with Turner syndrome remains obscure. Both primary and secondary Empty Sella Syndromes may be potential underlying causes, and some familial histories suggest the presence of an as-yet-undefined genetic-related patho-physiological mechanism that warrants further investigation.

## Introduction

Turner syndrome (TS) is a genetic disorder caused by the partial or complete absence of one X chromosome in females, with an estimated incidence of approximately 1 in 2,000 live female births. Empty Sella Syndrome (ESS) is radiologically defined by the herniation of cerebrospinal fluid into the sella turcica, resulting in compression of pituitary tissue. ESS is often associated with hypopituitarism, as well as various neurological and ophthalmological manifestations. The co-occurrence of TS and ESS is rare, and existing reports primarily describe TS patients with ESS presenting during childhood with growth retardation and delayed pubertal development. To the best of our knowledge, cases of TS complicated by ESS with multiple pituitary hormone deficiencies manifesting in the postpubertal period remain exceedingly uncommon. The underlying pathophysiological mechanisms remain poorly understood. This study aims to explore the potential association between TS and ESS through case reviews, thereby contributing to future research and informing clinical management strategies.

A comprehensive literature search was conducted using PubMed, MEDLINE, and manual screening of reference lists. The inclusion criteria were as follows: (1) articles published up to September 2024; (2) search terms including “Turner syndrome,” “empty sella syndrome,” “empty sella,” and their combinations; and (3) no restrictions applied regarding publication date. Exclusion criteria included non-English articles, review articles, abstracts lacking sufficient relevance, and studies for which the full text was not accessible. Ultimately, ten eligible case reports—including the present case—were identified and included in the final analysis (**Table 1**).

**Table 1:** Review of studies on Turner syndrome with empty sella syndrome.

Author (year)	Age at diagnosis (years)	Chief complaint	Concomitant Conditions	Chromosomal Karyotype	Pituitary MRI findings	Compromised Hormone Axes
Fanyu (2024)	29	Amenorrhea	–	45,X[9]/46,XX[21]	Partial empty sella	HPG; HPT; HPS
Dutta (2015) (1)	16	Short stature Delayed puberty	Hashimoto’s thyroiditis	45X0	Empty sella with ectopic Neurohypophysis near tuber cinerium	HPG; HPT; HPS
Bougacha-Elleuch (2016) (2)	31	Typical picture of turner syndrome	–	45X0 (8%)/46XX	Empty sella	HPG; HPT; HPS
Bougacha-Elleuch (2016) (2)	14	Short stature Delayed puberty	–	45X0 (23%)/46XX/47XXX (3%)	Empty sella	HPG; HPT HPS was not evaluated
Bougacha-Elleuch (2016) (2)	17	Short stature Delayed puberty	–	45X0	Hypoplastic pituitary	HPG; HPT HPS was not evaluated
Grossi (2012) (3)	2.4	Not mentioned	A group of multiorgan autoimmunity-related manifestations	[46],XX,der(2)t(2;10)(2pter → 2q37::10p13 → 10pter)[127]/45,X,der(2)t(2;10)(2pter → 2q37::10p13 → 10pter)[23]	Partial empty sella	–
Gallicchio (2003) (4)	7	Frontal headache Asthenia Delayed growth	–	45,X/46,XX	Empty sella	HPG; HPT; HPS
Nishi (2002) (5)	13	Short stature Infantile genitalia	–	45,X0	Empty sella	None
Weibel (2014) (6)	43	Premature ovarian failure	–	mosaic Turner syndrome	Pituitary macroadenoma then develops into empty sella syndrome	Gonadotropin deficiency and then recovery
Valenta (1984) (7)	27	Primary amenorrhea	Hashimoto’s thyroiditis	45,X0	Partially empty sella anteriorly	HPG
Valenta (1984) (7)	16	Short stature Pubertal delay Primary amenorrhea	Primary hypothyroidism	46,XX with Xpl1.3-Xpter	Partially empty sella with posterior displacement of the pituitary	HPG
Lomna-Bogdanov (2005) (8)	51	Short stature	–	45,X [18]/46,XX [272]/47,XXX [10]	Partially empty sella and pituitary hypoplasia	HPG; HPT; HPS; HPA
Lomna-Bogdanov (2005) (8)	43	Hypopituitarism	–	45,X [14]/46,XX [282]/47,XXX	Pituitary hypoplasia	HPG; HPT; HPS; HPA

HPA, Hypothalamic-Pituitary-Adrenal Axis; HPT, Hypothalamic-Pituitary-Thyroid Axis; HPG, Hypothalamic-Pituitary-Gonadal Axis.

HPS, Hypothalamic-Pituitary-Somatotropic Axis; -, not mentioned.

## Case presentation

A 29-year-old woman was admitted on September 2, 2024, with a 15-year history of irregular menstruation following menarche and 4 months of amenorrhea. She reported cold intolerance and denied any history of headaches, visual changes, or fatigue. There was no history of other drug use and no history of fractures. Her family and genetic history were normal. One month prior, outpatient evaluations revealed low levels of estradiol, follicle-stimulating hormone, and luteinizing hormone, with normal thyroid-stimulating hormone and decreased free thyroxine. Baseline cortisol and insulin-like growth factor 1 levels were also low. Prolactin levels were normal (**Table 2**). Liver function, renal function, serum electrolyte levels, oral glucose tolerance test with insulin measurements, and urine specific gravity were all normal (**Table 2**).

**Table 2:** Basal values of anterior pituitary hormones in this patient.

Parameter	July 4th, 2023	July 9th, 2023	Aug 14th, 2024	Oct 16th, 2024	Reference
TSH (mIU/L)	3.715	–	3.485	2.497	0.55-4.78
FT4 (pmol/L)	10.83	–	10.95	13.26	11.5-22.7
FT3 (pmol/L)	4.40	–	4.46	4.67	3.5-6.5
LH (IU/L)	6.4	6.9	4.3	9.1	–
FSH (IU/L)	2.7	5.0	6.1	6.6	–
E2 (pg/ml)	241.7	32.8	34.3	29.4	–
P(ng/ml)	0.47	0.56	0.27	0.31	–
IGF-1 (ng/ml)	–	–	85	–	167-669
IGF1-bp3 (ug/ml)	–	–	3.65	–	3.4-7.0
AMH (ng/ml)	–	3.39	–	4.11	–
PRL (ng/ml)	2.1	8.8	–	8.9	2.8-29.2
Cort-8am (nmol/l)	–	–	166.3	–	119-618
ACTH-8am (pg/ml)	–	–	8.59	–	<46

TSH, thyrotropin; FT4, free thyroxine; FT3, free triiodothyronine; LH, luteinizing hormone; FSH, follicle-stimulating hormone; E2, Estradiol; P, progesterone; IGF-1, Insulin-like Growth Factor 1; IGF1-bp3, Insulin-like Growth Factor 1 Binding Protein 3; AMH, Anti-Müllerian Hormone; PRL, Prolactin; Cort, Cortisol; ACTH, Adrenocorticotropic Hormone; -, not mentioned.

On physical examination, blood pressure was recorded as 119/80mmHg, height was 153 cm (mother’s height: 155 cm, father’s height: 172 cm), weight was 50 kg, and body mass index was 21.35 kg/m². There was mild cubitus valgus on the right hand, and breast development was Tanner Stage IV. External genitalia showed female distribution of pubic hair, classified as Tanner Stage IV. Color Doppler ultrasound attachment indicated: Uterine body anteroposterior diameter of 4.3 cm, endometrium is centrally located, endometrial thickness of 0.5 cm (single layer), with heterogeneous echogenicity, and normal-sized bilateral ovaries. Pituitary magnetic resonance imaging showed partial empty sella with pituitary compression (dimensions: 0.9 × 0.2 × 1.3 cm). Following admission, an insulin-induced hypoglycemia stimulation test indicated mild reduction in growth hormone axis function, while the cortisol axis showed compensatory function (**Table 3**). Chromosomal analysis revealed 45,X[9]/46,XX[21].

**Table 3:** Basal and insulin stimulated values of the patient.

Parameter	-30min	0min	30min	45min	60min	90min
Glucose (mmol/L)	4.76	1.14	4.8	4.9	4.3	3.83
Growth Hormone (ng/ml)	0.12	0.1	7.16	8.73	9.48	3.09
Cortisol (nmol/L)	166.3	156.3	362.3	368.0	400.4	547.3

Time points are referenced relative to the insulin intravenous injection at 0 minutes.

The patient was diagnosed with TS in association with ESS and anterior pituitary hormone deficiencies, involving the hypothalamic–pituitary–gonadal, –thyroid axes. Levothyroxine replacement therapy was initiated at a dose of 25 μg/day. An artificial menstrual cycle was induced using a combination of drospirenone and ethinylestradiol, during which the patient maintained regular menstrual bleeding. Follow-up laboratory evaluations in October 2024 demonstrated improved thyroid function; detailed results of thyroid and sex hormone profiles are presented in **Table 2**. Given her expressed desire for fertility, the patient was referred to the reproductive endocrinology department for further assessment, with plans to undergo preimplantation genetic testing for aneuploidy–assisted conception.

## Discussion

Turner syndrome (TS) is one of the most common sex chromosome abnormalities, occurring in approximately 1 in 2000 live female births (9). The clinical manifestations of TS are diverse and may include short stature, gonadal dysgenesis, webbed neck, cubitus valgus, congenital cardiovascular diseases, and an heightened incidence of autoimmune diseases (10). Due to primary gonadal dysgenesis, TS is typically associated with primary ovarian insufficiency and presents as hypergonadotropic hypogonadism, characterized by elevated levels of follicle-stimulating hormone and luteinizing hormone levels (11). However, the phenotypic spectrum of TS is broad; some individuals may undergo spontaneous menarche and exhibit initially regular menstrual cycles, leading to potential underdiagnosis. Empty sella refers to a radiological finding in which the sella turcica is partially or completely filled with cerebrospinal fluid, resulting in compression and flattening of the pituitary gland along the floor or sides of the sella. This condition is more frequently observed in individuals over 40 years of age, particularly in multiparous women with obesity and hypertension, and is relatively uncommon in pediatric populations. A minority of individuals with an empty sella may present with clinical symptoms such as headache, visual disturbances, visual field defects, endocrine dysfunction, hyperprolactinemia, oligomenorrhea, or amenorrhea, in which case the condition is referred to as Empty Sella Syndrome (ESS) (12).

The co-occurrence of TS and ESS is an uncommon clinical finding, and its true prevalence remains uncertain. In most reported cases, the diagnosis of TS concomitant with ESS was established based on characteristic features such as growth retardation and delayed pubertal development. To better understand this association, we reviewed and summarized the key clinical characteristics of all relevant cases reported in the literature between 1962 and 2024 (**Table 1**). Several studies (2, 7, 8) have suggested that this combination may represent a distinct clinical subtype. Notably, none of the reviewed cases demonstrated evidence of hypopituitarism after the completion of pubertal gonadal development. Based on both the existing literature and clinical observations, we propose the following differential diagnostic considerations:

1. Primary empty sella: primary ESS arises from a developmental defect or insufficiency of the diaphragma sellae, permitting herniation of the arachnoid membrane into the sella turcica through an enlarged aperture. This leads to compression and flattening of the pituitary gland. While it can manifest in childhood, pituitary compression may result in varying degrees of hypopituitarism. In our case, the patient had no history of cranial radiotherapy or neurosurgery, reported no headache symptoms, and exhibited a slowly progressive clinical course—features consistent with primary empty sella. This condition may stem from congenital weakness or malformation of the diaphragma sellae, which can deteriorate over time due to growth or fluctuations in intracranial pressure, ultimately leading to pituitary dysfunction. Previous reports by Bougacha-Elleuch et al. (2) and Lomna-Bogdanov et al. (8) described familial cases of TS associated with empty sella and multiple pituitary hormone deficiencies, suggesting a potential shared genetic basis. These findings raise the possibility that genetic factors linked to TS may contribute to the development of primary empty sella. For example, haploinsufficiency of the SHOX gene is known to underlie the characteristic skeletal abnormalities and short stature in TS patients (13). Based on these considerations, we recommended genetic testing for our patient to evaluate the integrity of X-linked genes and explore whether gene dysfunction might contribute to sella turcica hypoplasia. However, the patient declined genetic testing due to financial and social constraints. Notably, previously reported cases typically involved delayed pubertal onset, implying that pituitary dysfunction and empty sella may have originated in childhood or early adolescence. In contrast, our patient demonstrated established gonadal development, suggesting that pituitary dysfunction likely emerged after the onset of puberty, distinguishing her case from the aforementioned familial presentations.
2. Secondary empty sella: secondary ESS results from herniation of the arachnoid membrane into the sella turcica following events that compromise pituitary volume. These include space-occupying lesions (e.g., pituitary adenomas), repeated pregnancies, pituitary infarction, or therapeutic interventions such as surgery or radiotherapy involving the sellar region, as well as chronically elevated intracranial pressure. A case reported by Weibel et al. suggested that, in the postpubertal period, impaired gonadal function can lead to increased secretion of follicle-stimulating hormone and luteinizing hormone, which may induce reactive pituitary hyperplasia. It has been hypothesized that subsequent ischemic insult or infarction secondary to pituitary enlargement could underlie the development of secondary ESS (6). In our patient, however, no symptoms indicative of acute pituitary events—such as headache or visual field disturbances—were reported, and there are no historical records of gonadotropin levels or previous pituitary imaging for longitudinal comparison. Although the possibility of silent pituitary apoplexy cannot be definitively excluded, we lack direct evidence to support this diagnosis. Moreover, TS is frequently associated with autoimmune disorders, including autoimmune hypophysitis, which can also lead to pituitary dysfunction and ESS (7). Unfortunately, due to limitations in laboratory capabilities, pituitary autoantibody testing was not performed in this case. As such, autoimmune hypophysitis cannot be definitively ruled out and should remain a consideration in the differential diagnosis.

In summary, the majority of individuals with TS typically present with short stature, gonadal dysgenesis, and hypergonadotropic hypogonadism. Our case, along with previous reports, highlights the rare coexistence of TS and ESS, with some cases also demonstrating additional features such as growth hormone deficiency, hypogonadotropic hypogonadism, and, in exceptionally rare instances, central adrenal insufficiency. While several studies have proposed potential shared pathophysiological mechanisms—such as genetic predisposition or autoimmune-mediated processes—the current body of evidence remains limited and inconclusive. Given the complex and multifactorial etiology of ESS, any suggested association with TS should be interpreted with caution. Further research is necessary to elucidate the underlying mechanisms and to determine whether a causal relationship exists.

## Conclusions

TS combined with ESS has been reported in some literature, but it remains rare, particularly in patients who exhibit multiple pituitary hormone deficiencies after gonadal development. As a result, such cases are often overlooked and underdiagnosed. We believe that when empty sella or other central nervous system abnormalities are present, a thorough evaluation of pituitary function is warranted.

## Data Availability

The raw data supporting the conclusions of this article will be made available by the authors, without undue reservation.
